# Prevention and treatment of urinary tract infection with probiotics: Review and research perspective

**DOI:** 10.4103/0970-1591.40604

**Published:** 2008

**Authors:** D. Borchert, L. Sheridan, A. Papatsoris, Z. Faruquz, J. M. Barua, I. Junaid, Y. Pati, F. Chinegwundoh, N. Buchholz

**Affiliations:** 1 Department of Urology, St. Bartholomew's Hospital, London, UK; 2 Department of Microbiology, St. Bartholomew's Hospital, London, UK; 3 Department of Urology, Barking, Havering and Redbridge Hospitals, London, UK; 4 Department of Urology, Homerton University Hospitals, London, UK; 5 Department of Urology, Newham University Hospitals, London, UK

**Keywords:** Prevention, probiotics, urinary tract infection

## Abstract

The spiralling costs of antibiotic therapy, the appearance of multiresistant bacteria and more importantly for patients and clinicians, unsatisfactory therapeutic options in recurrent urinary tract infection (RUTI) calls for alternative and advanced medical solutions. So far no sufficient means to successfully prevent painful and disabling RUTI has been found. Even though long-term oral antibiotic treatment has been used with some success as a therapeutic option, this is no longer secure due to the development of bacterial resistance. One promising alternative is the use of live microorganisms (probiotics) to prevent and treat recurrent complicated and uncomplicated urinary tract infection (UTI).

The human normal bacterial flora is increasingly recognised as an important defence to infection. Since the advent of antibiotic treatment five decades ago, a linear relation between antibiotic use and reduction in pathogenic bacteria has become established as medical conventional wisdom. But with the use of antibiotics the beneficial bacterial flora hosted by the human body is destroyed and pathogenic bacteria are selectively enabled to overgrow internal and external surfaces. The benign bacterial flora is crucial for body function and oervgrowth with pathogenic microorganisms leads to illness. Thus the concept of supporting the human body's normal flora with live microorganisms conferring a beneficial health effect is an important medical strategy.

## GENERAL REMARKS ON PROBIOTICS

Epidemiological evidence is an important reason to support research on alternative treatment options. There is a epidemiological evidence on significant problems with multiresistant bacteria (bacteria resistant to multiple antibiotics) like *Clostridium difficile (C. difficile)* and methicillin resistant *Staphylococcus aureus* (MRSA) in the UK and elsewhere.[1–3] The development of bacterial resistance relies on several factors. One of these is the widespread use of antibiotics. Frequent use of quinolones in urology departments may contribute to the outbreaks in antibiotic associated *C. difficile* diarrhoea.[4] Alternative therapeutic options should use strategies to (a) prevent the selective development of antibiotic resistant bacterial strains, (b) restore a balanced microbial flora and (c) enhance the defence mechanisms of the human body. These criteria are best fulfilled by live microorganisms which are naturally hosted by the human body already. Positive and convincing effects have already been shown, e.g., in reducing complications after major abdominal surgery and acute and chronic diarrhoea.[5–8] A recent report on the use of probiotics in antibiotic-associated diarrhoea underlines that this is possible already with commercially available probiotic drinks.[9] So far, no sufficient trials have been undertaken to support the use of probiotics in patients with RUTI.

## CONCEPT OF PROBIOTICS

The bacterial flora of the skin and mucosal surfaces is an important barrier to infection. Within the normal bacterial flora host defence is ensured through a balance between non-pathogenic commensals and pathogenic bacteria. It is well established that in the immunocompromised host as well as with antibiotic treatment the natural protective biofilm of bacteria and surface-cells is disrupted. In this regard, it is interesting to note that, for example, in HIV + patients lactobacillus colonisation of the urogenital tract is diminished and this correlates with the shedding of HIV into the tract.[10] Disruption of the natural flora renders patients prone to severe infection with not only one, but also several pathogenic microorganisms. A strategy to restore a host-supportive bacterial flora involves the use of probiotics. Probiotics are defined as live microorganisms which when administered in adequate amounts confer a health benefit to the host.[11] There are two main scientific concepts associated with probiotics. Live microorganisms administered orally or applied to the genital area overgrow pathogenic flora and restore an environment resistant to infections (competitive theory). This has been impressively demonstrated in an infection model with *Giardia intestinalis* and verified by studies on the human microflora.[12,13] The competitive concept has achieved a breakthrough in acute diarrhoea with the publication of a meta-analysis of 34 masked, randomised, placebo-controlled trials, showing a definitive benefit from treatment with probiotics.[14] Moreover, the benign bacterial flora produces different metabolites and these are directly bactericidal or bacteriostatic to pathogenic flora in the same host.[15,16] Individual strains of live microorganisms have been found to elicit specific inhibitory capacities on the growth of problem bacteria like MRSA and *C. difficile*.[17–20] Moreover, probiotics can exhibit a synergistic effect with antibiotics.[21]

The second, controversial theory is based upon modulation of the immune system.[22] Live microorganisms are known to influence production of immunoglobulins and thus altering the body's immune defence. They are also able to contribute to a specific immune response against pathogenic bacteria.[12]

### Probiotics are safe to use

Probiotics can be regarded as safe according to a report of the Central Public Health Laboratory, London (now the Health Protection Agency Centre for Infections).[23] Epidemiological studies confirm no increase in bacteriaemia due to probiotic medication after nationwide introduction in Finland.[24] Especially, Lactobacilli have GRAS status (generally safe to use).[25] As it seems somewhat counterintuitive to use one sort of bacteria to fight another sort of bacteria and bacteria are generally seen as pathogenic, it is no surprise that the safety of probiotics have been carefully monitored and investigated.[26,27] Rarely, cases like a liver abscess caused by *Lactobacillus rhamnosus* have been reported.[28] Recently, case reports on infections from clinical use of probiotics have extensively been reviewed.[29] As a result, the authors agree that probiotics are generally safe but should be used cautiously in immunocompromised patients. In this review of case reports, it is also sensibly pointed out that safety must be established for each individual strain used in probiotic preparations. Trautner *et al*. write in their report on *E. coli* HU2117 coated urinary catheters that “the potential pitfall of bacterial interference is that no living organism is truly avirulent in an immunocompromised host”.[30] Despite having no side effects in their 12 patients studied and despite cautious views from others probiotics have been successfully trialled in immunocompromised patients.[5,31,32]

### Probiotics are already widely used

Probiotics are already widely used as over-the-counter drugs, including in yoghurts and probiotic drinks. However, their status is similar to herbal medicine. Currently, over-the-counter probiotics are regarded as food supplements. These types of probiotics have low counts of live microorganisms compared to probiotic preparations studied in clinical trials. Probiotics are used in individual centres as an additional treatment in a variety of chronic diseases. In many instances, probiotics have been trialled in small numbers of patients to gain experience in their use, safety and efficiency. But in Finland, Sweden, Denmark, Germany and the Czech Republic, probiotics have been tested in several thousands of adults and in infants in observational studies over 20 years.[33,34] The probiotic fermented milk drink Yakult has been sold in Japan since 1935 according to Hoesl and Altwein.[35] Many of these microorganisms are traditionally used by humans for food production, like the fermentation of meat, cheese and beverages.[36,37] The idea of using these microorganisms for medical treatment has existed for many years. Evidence from laboratory research as well as from clinical trials exists to demonstrate the therapeutic effects of live microorganisms, if used in appropriate dosage and setting.

### Economic reasoning for research on alternative strategies

Urinary tract infection can be regarded as one of the most common community-acquired, hospital-acquired and recurrent types of infection. In the United States, UTIs result in US $1.6 billion in healthcare cost each year.[38] Costs associated with RUTI have not been assessed on a national basis in the UK so far. As infections of the urogenital tract are the most common type of infection worldwide, it can be extrapolated from the US data that treatment of UTI has a major impact on the NHS. In the UK more than 320,000 patients develop infections while in hospital each year and this leads to more than £900 million in costs. Probiotics are a potentially cheap alternative to prevent a large share of these hospital-acquired infections.[39] More importantly clinical trials with probiotics are not expected to increase treatment costs for the participants as has been shown in even more complex oncological patient groups.[40]

## TRIALS ON EFFICACY

### Trials with probiotics in diseases other than RUTI

Investigation and trials with probiotics have so far covered a wide range of diseases and included the prevention and treatment of caries and tonsillitis[41,42]; gastrointestinal disease like acute and chronic diarrhoea, irritable bowel syndrome and *Helicobacter pylori* infection, as well as in the immunocompromised host with drug-associated diarrhoea.[21,31,32] Randomised-controlled trials and prospective investigations have been performed in critically ill patients with acute pancreatitis and in major abdominal surgery.[5,43] To date there is supporting but not sufficient data to generally recommend the use of probiotics in critically ill patients and those undergoing major surgery, although results of recent randomised trials have been very encouraging.[44,45] For different types of diarrhoea, sufficient data are now available resulting in repeated meta-analysis.[7,8,46–48] This allows the targeted clinical use of probiotics in antibiotic-associated and travellers diarrhoea. Antibiotic-associated *C. difficile* positive diarrhoea is a problem to all medical specialities and so it is for urology. Therefore, off definitive interest for urologists is the finding that not only clinical preparations of specific probiotics, but also commercially available probiotic preparations like probiotic drinks are effective in preventing and treating this type of diarrhoea.[9] From these trials, definitive recommendations can be given for the use of probiotics in acute and chronic diarrhoea.[7] Again of significant interest for the urologist within the multidisciplinary care and treatment of prostate cancer patients is the finding that probiotics are effective in preventing radiation-induced diarrhoea.[49]

### Trials with probiotics

Trials on the use of probiotics in urology patients to date had small numbers of participants only. There are small studies on the use of probiotics in renal calculi due to enteric hyperoxaluria, recurrent candida vulvovaginitis, as well as UTIs.[50–52] In patients with neurogenic bladder trials with encouraging results have been performed with instillation of non-pathogenic *E. coli* into the bladder.[53,54] To date two clinical trials are on the way to explore the effects of oral and topical probiotics in RUTI.[55,56] No trials in this area have been started or performed in the UK.

The RUTI is a significant healthcare problem worldwide for many women and even more so in specific patient populations. Patients with spinal cord injury and neurogenic bladder as well as patients with long-term urinary catheter all share the problem of RUTI. These patients do have more complicated UTI and develop resistance to standard antibiotics. The recent reports on MRSA, *C. difficile* and other problem pathogens in the UK leave no doubt that alternative, preventive and economic therapeutic options to antibiotics are urgently needed.

The use of oral probiotics has not been sufficiently tested in RUTI and they have not been tested at all in patients with neurogenic bladder or long-term urinary catheter. Recently, Darouiche *et al*. tested the topical use of probiotics in patients with neurogenic bladder. After instillation of a benign *E. coli* strain into the bladder of these patients, they found decreased rates of RUTI especially in those, where the bladder was successfully colonised.[57] The same group started to look at urinary catheters coated with probiotic microorganisms in contrast to catheters coated with antimicrobials. Twelve adult inpatients with neurogenic bladders requiring indwelling urinary catheters had *E. coli* HU2117-coated catheters inserted for 28 days. With this method, the rate of symptomatic UTI was reduced to 0.15 cases per 100 patient-days compared to published average rates of 2.72 cases per 100 patient-days in such patients.[30] In women, the topical use of Lactobacilli released from a vaginal suppository has been investigated in a pilot trial in nine women. It was shown that *E. coli* positive cultures reduced from 5.0 ± 1.6 episodes to 1.3 ± 1.2, *P* < 0.0007 over 12-month period.[58] The cited studies did not report any serious side effects or intolerance, but suggested that severely immunocompromised hosts may only be trialled with caution.

A trial with oral probiotics is currently under way in the Netherlands (NAPRUTI trial) using different strains of oral probiotics, containing *L. rhamnosus* and *Lactobacillus reuteri*.[55] In this multicentre double blind trial, 280 postmenopausal women are randomised to receive either oral Lactobacilli or standard antibiotic treatment for RUTI. Patients are treated for 12 months with a follow-up of 3 months. Another trial in the United States investigates the use of a topical single strain probiotic with *Lactobacillus crispatus*.[56] This single centre trial investigates uncomplicated RUTI in premenopausal women only. A total of 100 female patients are randomised to receive either placebo or topical Lactobacilli as a vaginal capsule for 3 months with a follow-up of 6 months. Neither trial compares premenopausal to postmenopausal treatment with probiotics. Moreover, probiotics are not expected to completely eradicate infections but to lower the rate of recurrence and prevent development of bacterial resistance. In this regard, the trial designs do not describe precautions or scenarios on the use of probiotics in episodes of UTI severe enough to require additional treatment.

Probiotics can be regarded as the single most powerful alternative option under clinical development for the prevention and treatment of chronic infection.[59] Given the enormous burden on patients, as well as the scientific and economic problem caused by RUTI, the investigation of probiotics is of potentially crucial importance for patient benefit and clinical science. Laboratory and clinical research on live microorganisms have opened a major research field with increasing numbers of investigations and trials. Little is known about the complex interaction of the human bacterial flora with the human body. From an evolutionary point of view, live microorganisms have provided the human body with crucial functions in digestion and immunemodulation. The human body did not have to develop these functions and is employing the hosted flora of microorganisms “as a metabolic ‘organ’ exquisitely tuned to our physiology” on its outer surfaces.[60] The bacterial flora of the gut has a weight of approximately 1-2 kg and is thought to be metabolically as important as the liver.[61] As the live microorganisms used in probiotics are often isolated from the human flora, trials with specific probiotics will help to elucidate the role of these bacteria in the human body's eco-system. Data and experience gained from clinical trials with probiotics will direct laboratory research and help to train clinicians in their future clinical use.

The harmful effects of antibiotics have always been somewhat overlooked. The scientific importance of trials with probiotics is not only to investigate their potential use in recurrent infection, but also the containment and therapy of the side effects of antimicrobial chemotherapy itself.

A major concept in urological therapy is to prevent the recurrence of UTI. Investigations on live microorganisms derived from the human gut flora will drive forward the field of preventive medicine in the therapy of RUTI. Similar to nutritional aspects in medicine probiotics acknowledge the complex nature of infection. Despite longstanding knowledge of immunosuppressive effects of poor nutrition, the introduction of perioperative enteral nutrition has only recently been developed.[62] Perioperative enteral nutrition has a major impact on the body's ability to resist infection. This view and treatment strategy has now been added to antibiotic therapy for infection in most surgical specialties, giving evidence of the need for complementary anti-infective prevention and treatment.[63,64] As described above, despite definitive clinical evidence on the positive effects of probiotics, so far sufficiently powered studies using probiotics in RUTI have only recently been commenced.[65,66]

### Bladder cancer - another reason to trial probiotics

Hoesl and Altwein recently reviewed the impact probiotics could have on bladder cancer therapy.[35] With Bacillus Calmette-Guerin (BCG) immunotherapy as the gold standard for prevention of the recurrence of superficial bladder cancer, the urologists have actually been for a long time at the forefront in using “microorganisms” for therapy. Thus it seems very reasonable to trial other microorganisms in urological disease as well. In 2002, Ohashi *et al*., reported in a case-control study in 180 patients on the habitual intake of lactic acid bacteria, suggesting that these microorganisms are able to prevent the development of bladder cancer.[67] From the early 1980s on many experimental studies have shown potential mechanisms whereby probiotics could prevent bladder cancer, including inhibition of carcinogens and their cytotoxic effects as well as local and systemic modulation of the immune response.[68] At least two clinical trials found probiotics to be effective in the treatment of superficial bladder cancer.[69,70] Hoesl and Altwein as many others have stated that probiotics are cheap and non-toxic, compared to many chemotherapeutic agents. This makes probiotics ideal candidates in cancer prevention and treatment trials.

## CONCLUSION

To date insufficient data exists to support the routine use of probiotics in urological diseases such as RUTI or bladder cancer. But probiotics show promise in becoming an alternative or complementary treatment option for many diseases. As probiotics are already in use in many fermented products, there are no major safety concerns. Thus it is probably only the targeted use of these microorganisms which has to be learnt from clinical trials. Probiotics are derived mainly from the human gut flora and belong to a still poorly understood metabolic organ of the human body. Trials on probiotics would help to understand this metabolic organ and use it to counterbalance traditional antimicrobial chemotherapy. Probiotics have the potential for a future alternative prevention and treatment strategy in RUTI. They are also potentially preventive for cancer development and progression. In conclusion, research on the field of live microorganisms advances scientific knowledge on (a) the clinically significant problem of RUTI, (b) on the prevention and treatment of infection in general, (c) on the understanding of the function of the bacterial eco-system within the human body and (d) on the collateral effects of antimicrobial chemotherapy.
